# The role of previously undocumented data in the assessment of medical trainees in clinical competency committees

**DOI:** 10.1007/s40037-020-00624-x

**Published:** 2020-10-06

**Authors:** Jennifer Tam, Anupma Wadhwa, Maria Athina Martimianakis, Oshan Fernando, Glenn Regehr

**Affiliations:** 1 Centre for Health Education Scholarship, Faculty of Medicine, University of British Columbia, Vancouver, British Columbia Canada; 2 Division of Infectious Diseases, Department of Pediatrics, University of British Columbia, Vancouver, British Columbia Canada; 3 Division of Infectious Diseases, Department of Pediatrics, University of Toronto, Toronto, Ontario Canada; 4 Wilson Centre for Research in Education, University of Toronto, Toronto, ON Canada; 5 Department of Paediatrics, Faculty of Medicine, University of Toronto, Toronto, ON Canada; 6 Department of Paediatrics, The Hospital for Sick Children, Toronto, ON Canada; 7 Department of Surgery, University of British Columbia, Vancouver, British Columbia Canada

**Keywords:** Clinical competence committees, Undocumented data, Data management, Group decision-making, Competency-based medical education

## Abstract

**Introduction:**

The clinical competency committee (CCC) comprises a group of clinical faculty tasked with assessing a medical trainee’s progress from multiple data sources. The use of previously undocumented data, or PUD, during CCC deliberations remains controversial. This study explored the use of previously undocumented data in conjunction with documented data in creating a meaningful assessment in a CCC.

**Methods:**

An instrumental case study of a CCC that uses previously undocumented data was conducted. A single CCC meeting was observed, followed by semi-structured individual interviews with all CCC members (*n* = 7). Meeting and interview transcripts were analyzed iteratively.

**Results:**

Documented data were perceived as limited by inaccurate or superficial data, but sometimes served as a starting point for invoking previously undocumented data. Previously undocumented data were introduced as summary impressions, contextualizing factors, personal anecdotes and, rarely, hearsay. The purpose was to raise a potential issue for discussion, enhance and elaborate an impression, or counter an impression. Various mechanisms allowed for the responsible use of previously undocumented data: embedding these data within a structured format; sharing relevant information without commenting beyond one’s scope of experience; clarifying allowable disclosure of personal contextual factors with the trainee pre-meeting; excluding previously undocumented data not widely agreed upon in decision-making; and expecting these data to have been provided as direct feedback to trainees pre-meeting.

**Discussion:**

Previously undocumented data appear to play a vital part of the group conversation in a CCC to create meaningful, developmentally focused trainee assessments that cannot be achieved by documented data alone. Consideration should be given to ensuring the thoughtful incorporation of previously undocumented data as an essential part of the CCC assessment process.

**Electronic supplementary material:**

The online version of this article (10.1007/s40037-020-00624-x) contains supplementary material, which is available to authorized users.

## Introduction

The clinical competency committee (CCC) is considered an essential component of resident assessment in competency-based medical education [1–5]. While the format of a CCC varies between training programs, it is typically composed of faculty tasked with the complex process of synthesizing trainee performance data from multiple sources [5, 6] to collaboratively determine each trainee’s readiness to proceed to the next stage of training and to construct a plan for subsequent educational experiences [1, 5, 6]. Depending on the size of the program, some CCCs may include a large proportion of the faculty, while others comprise a small subset of the faculty such that very few members on the committee may have worked directly with particular trainees being discussed. Although some training programs have utilized CCCs for some time [7], the use of previously undocumented data (or PUD) during CCC deliberations remains controversial. For instance, while the Accreditation Council of Graduate Medical Education of the United States acknowledges that knowledge gained from informal previously undocumented sources can be useful [5], the Royal College of Physicians & Surgeons of Canada (RCPSC) has favoured the opposite view, stressing that only data formally documented prior to the meeting should be used to inform discussions [6].

Clearly, high-stakes promotion decisions by the CCC must be defensible; decisions based on hearsay or rumor may render CCC decisions unsupportable in appeals processes [8]. Additionally, concerns have been raised that the use of previously undocumented data in CCC meetings might distort trainee assessments [9]. However, effective documentation of clinical performance remains elusive [10]. For example, Ginsburg and colleagues found that preceptors’ subjective impressions of a resident’s performance often did not fit a typical competency framework, suggesting that standardized scales might constrain authentic depictions of residents’ performance [11]. Further, while there may be additional value in written narratives [12–15], these may still not provide a complete picture due to raters’ “saving face” efforts [16] and their use of vague language [17]. In part, these limitations of documentation likely arise from social barriers, such as raters being hesitant to formally document negative trainee assessments unless there is evidence that their colleagues hold similar opinions [10, 18, 19]. CCC discussions using previously undocumented data may overcome at least some of these limitations of documented assessment data. For example, research suggests that CCCs may identify residents in difficulty earlier if previously undocumented data are used [20], and that the collective use of previously undocumented data may yield richer assessments [21].

In short, while concerns about the potential misuse of previously undocumented data are important to attend to, it is not well understood when these data might be important, how such data are introduced and handled by a committee, or how they influence the decision-making process. In fact, we have found no clear definition or taxonomy of previously undocumented data in the literature. As training programs begin to adopt the formalized structured process of CCCs, understanding how to best balance previously undocumented data with documented data may be useful in developing more meaningful policies with regard to their use. The purpose of this study was to explore the kinds and purposes of previously undocumented data that are invoked in a CCC, and how undocumented data are incorporated with documented data (including both numerical and narrative comments) to create an impression of a trainee’s competence in the setting of CCC assessments.

## Methods

### Design

We employed an instrumental case study methodology [22] with the goal of identifying patterns and developing taxonomies that might inform current assumptions regarding the use of previously undocumented data in CCCs. Instrumentally, we defined previously undocumented data as any contribution to the meeting intended to aid in trainee assessment that was not available in any documented data brought to the meeting. Data were collected via observations of a CCC meeting followed by semi-structured interviews of the committee members. Ethical approval was obtained through the Hospital for Sick Children’s Research Ethics Board (REB Number: 1000059941; date of approval: November 14, 2018).

### Setting

The CCC under study was that of a two-year postgraduate medical subspecialty training program in Canada, which has employed a form of CCC as part of its resident assessment system since 2008. The program admits approximately 3–4 trainees per year (6–8 trainees in total), including Canadian Medical Graduates and International Medical Graduates. CCC membership included all seven faculty within the division, including the division chief and the program director. Most of the CCC members have served on the committee for its entire existence. Given the relatively small size of the program, each CCC member typically has had the benefit of individual supervisory experience with each trainee prior to the CCC meeting.

The CCC currently meets twice a year with all clinical faculty present. Since 2017, a faculty member other than the program director serves as chair of the CCC to better align with the RCPSC guidelines. The information generated from the CCC meeting includes both a promotion decision and feedback to inform future training. The program director takes notes during the meeting, documents the results of the discussion, and shares the feedback with the trainee at his/her next scheduled meeting.

### Subjects

All members of the CCC agreed to participate in the study. Informed consent for the observation and interviews was obtained at a Division Meeting prior to the observed CCC meeting by O.F., who did not have a prior affiliation to the program. Participants did not receive any compensation for participation.

### Data collection

Data collection occurred from January to February 2019. A field observation of a CCC meeting was performed by O.F. and J.T. to track the use of previously undocumented data. The audio-recording from the meeting was transcribed verbatim. Observation notes were used to inform and refine the subsequent interview guide.

In-depth, individual, semi-structured interviews with each CCC member were conducted by O.F. and/or J.T. to explore the perceptions of participants with regard to the use of previously undocumented data (Appendix 1 of the Electronic Supplementary Material). Interviews and analyses were conducted iteratively, enabling modifications of the interview guide based on early insights and findings of interest. Each interview was audio-recorded and transcribed verbatim.

### Data analysis

Data collection and analysis occurred iteratively from January to April 2019. Through a close reading of observational and interview texts, we looked for patterns in the use of previously undocumented data. Initial codes and preliminary categories were discussed with all members of the research team prior to being used to code all of the transcripts. When new codes and categories emerged, these were discussed amongst the group. Minor modifications to the interview guide were made as findings transpired. NVivo software (version 11, QSR International, Australia) was used to facilitate data coding.

Study rigor was enhanced by the use of method triangulation (observation and interviews) and investigator triangulation. G.R., O.F., and M.A.M. were PhD-trained experts in medical education but outsiders to this CCC. J.T. was a former trainee in the program and A.W. was the program director at the time of the study. These insider perspectives supported our ability to “make sense” of the observation and interview findings, enhancing the rigor of the analysis. We specifically asked members of the CCC if there were any differences between this meeting and previous meetings, and the unanimous response was that this meeting was typical of previous meetings; therefore, it seems that any increased sensitivity to the use of previously undocumented data did not affect A.W.’s engagement in a way that was noticeable by the members. Additionally, an audit trail for decisions during data analysis and detailed reflexivity through journaling were used.

## Results

Six trainees were discussed at the observed 90-minute meeting. Individual semi-structured interviews were conducted with all six committee members who were present at the meeting (including one key informant interview). One committee member was away on leave at the time the CCC meeting was observed, but still participated in the semi-structured interview, resulting in seven interviews in total.

The observed meeting was felt by participants to be generally representative of a typical meeting. Discussion of each trainee began with a “formal” presentation of relevant information about the trainee. This started with a 2 to 4 minute oral presentation given by the trainee’s assigned mentor within the Division. This included details felt to be immediately relevant or pressing for the trainee, such as plans following subspecialty training, pertinent personal contexts, and a general sense of the trainee’s progress. The mentor report was followed by a recitation by the meeting chair of the formally documented record (clinical rotation evaluations, presentation evaluations, annual written exam scores, and research status report), which was largely numerical with limited narratives. Following this initial formal structure, the chair opened the floor to comments and discussion from the full committee, which typically took approximately 10 minutes.

Within this structure, previously undocumented data emerged in three contexts. First, the mentor’s report was based on the mentor’s overall knowledge of the trainee from personal conversations and interactions. Although the mentor report was not documented ahead of the meeting, these data were generally regarded as “factual” and *could* have been presented as a formal document. Second, the period of relatively unstructured group discussion following the formal presentation often involved the introduction of undocumented data that were based on personal “insider” knowledge by individual committee members as a natural part of the discussion. Third, previously undocumented data sometimes manifested as spontaneous interruptions to the formal presentation prior to the start of the group discussion period. The previously undocumented data occurring in these last two contexts could be perceived as the more contentious type of data within the context of CCCs, and did not differ in form or function, so we will focus the remainder of our results on these two latter contexts but will not distinguish between them in our descriptions.

### Types of previously undocumented data

During observations of the meeting, we saw undocumented data taking four general forms: summary impressions, descriptions of contextualizing factors, personal anecdotes and hearsay.

#### Summary impressions

Often, committee members offered their own gestalt of a trainee’s progress through their own accumulated personal experiences working with them. For example, in discussing one trainee, a committee member stated:

*Even though she still has some holes in her basic knowledge, she’s now just a lot better than when she started last year, and she’s a great clinician. (CCC observation)*

Such comments were not based on a collection of documented experiences (that is, these comments did not arise through a review of the documentation), but rather appeared to draw on the impressionistic summary of undocumented experiences between the trainee and the speaker.

#### Descriptions of contextualizing factors

Also prominent in previously undocumented data statements were the presentation of trainee-related contextual factors that might not be widely known to other committee members. These comments provided context for interpretation of assessment data as well as for planning for the trainee’s next steps. Sometimes these contextual factors were specific logistical or practical challenges related to training, as with this committee member’s statement during a discussion of a trainee’s research progress:

*She had an upcoming research block in January and her plan was to get re-engaged with [her research], but … that coincided with her [leave]. (CCC observation)*

However, these factors could also include broader issues such as a trainee’s current interests and future goals (such as plans to return to their country of origin to practice).

#### Personal anecdotes

Less commonly, committee members offered more concrete examples of their experiences with trainees, making comments such as:

*[I asked] her a simple question, like, ‘What is the mechanism of resistance of Streptococcus pneumoniae to penicillin?’ and she did not know. (CCC observation)*

#### Hearsay

Twice, we heard a committee member offer information based on what others had said rather than their own direct knowledge. Once, such hearsay was used as supporting evidence during a discussion exploring a learner’s pattern of behaviour; while discussing a trainee’s pattern of being overly diligent a committee member stated:

*It’s interesting that there are some recent [specialty] grads that I know, and when they talk about him, they say he was exactly the same way in [the specialty] so this isn’t a [subspecialty] thing. (CCC observation)*

The other use of hearsay was invoked to offer insight regarding a topic that would otherwise be difficult for committee members to directly assess. In this instance, a committee member shared feedback about the trainee’s teaching skills that was provided as a verbal comment from a junior member of the team.

### Reasons for invoking previously undocumented data

In subsequent interviews, participants suggested that while putting various types of documented data together created a more complete picture, there was still a perceived need for information beyond the formal data to generate a meaningful assessment. All participants relayed that the limitations of documented data alone in assessing a trainee’s competence made previously undocumented data vital in the context of a CCC. As one participant stated:

*You can get an idea from the ITERs [in-training evaluation reports] … that someone is competent, fulfils CanMEDS roles. Doesn’t necessarily mean that they are in progress to becoming a [subspecialty] consultant. You’d need a more whole picture which included a more fulsome, real-life, practical view on the person’s strengths and weaknesses. … [T]hat’s the sort of thing you might discuss better in the meeting. (Interview 6)*

Concerns provided by participants during interviews regarding the use of documented information alone included inconsistent rater usage of assessment scales, limited time in assessors’ schedules to provide detailed assessments, and raters’ reluctance to document anything negative about a trainee due to fears regarding the permanency of official university documents and perceived limitations of their individual perceptions.

Within the meeting itself, previously undocumented data were brought up to achieve three broad purposes: to raise new issues, to enhance a growing collective impression or to counter a growing collective impression.

#### To raise new issues

The open discussion period enabled committee members to introduce previously undocumented data in order to raise issues that were not previously addressed during the formal structure. For example:

*I’ve been trying to teach him that sometimes there’s maybe more than one way to do things … Sometimes, he gets upset with the team when they’re not doing what he says … I don’t know if anyone else found that, but I found he can get intense sometimes. (CCC observation)*

The raising of new issues appeared to serve a dual purpose: to contribute to the assessment of the trainee by offering an individual’s impression to be discussed within the group, but also to provide an opportunity for the individual to check his/her opinion against others. These “accuracy checks” were often signalled by phrases such as “maybe it’s just me” or “I don’t know if anyone else found that”. During interviews, participants expressed that being able to receive group feedback on their own impressions of trainees was a useful unintended consequence of the CCC group meeting to better understand how to interpret their own assessments of trainees, particularly negative impressions—that is, to help distinguish between a personality conflict, a one-off event, or a true problem:

*[I]t is good to periodically evaluate and discuss the trainees, so that you can get other people’s viewpoint as well. Because, sometimes you wonder, is it just a bad day they had with you, or is it something specific to you or the interaction you had, or is it something that’s more generalizable … Because, you know, everybody has bad days and you don’t want to unfairly evaluate a trainee based on a one-off particular circumstance. (Interview 5)*

When taken up by the group as a common issue, however, introducing a new issue was beneficial for developing a more holistic picture of the issue at hand by providing a starting point for committee members to subsequently enhance or counter the opinion with their own impressions and experiences, as further described below.

#### To enhance a growing impression

Frequently, previously undocumented data served to enhance or reinforce a growing group impression. The initial seed of the impression stemmed from documented data or new issues raised as described above. The opportunity to draw from others’ experiences was particularly helpful when the issue raised was more vague in nature. For instance, using the example above of the trainee felt to be intense sometimes, a committee member responded:

*I was with him over the holidays … I think that I was trying to put my finger on it too when I was working with him … [example given of clinical patient encounter to illustrate how trainee was uncomfortable with a less aggressive treatment plan]. I think that’s similar to what you’re talking about … that dealing with uncertainty is something to work towards. I think it’s similar to what you’re saying, accepting grey areas, right? (CCC observation)*

In this specific example, the combination of multiple CCC members’ impressions illuminated that this intensity was rooted in discomfort with uncertainty rather than rudeness. Without the group’s amalgamation of experiences in different contexts, however, the same conclusions may not have emerged.

#### To counter a growing impression

Previously undocumented data were also used to counter a growing impression whenever a committee member felt that the presented data, either in documented or undocumented form, differed from their own sense of a trainee’s progress. The use of previously undocumented data to counter a growing impression was the most common form of interruption to the formalized process, with the intention to facilitate interpretation of documented data:

*[Regarding lower exam score than expected] That’s worth a closer look because I’m surprised that there are that many gaps because certainly, with her on clinical service over the last year, I’ve certainly seen major improvements just in terms of content … there might be language issues there? (CCC observation)*

However, differing viewpoints also enabled dialogue to occur between members during the open discussion period that would not have been feasible with individually completed documented assessments. For instance, after one committee member commented that a trainee’s letters seemed too long, other committee members reflected on their own experiences and commented that they did not have the same impression: “*Oh, I thought they were fine, what I can remember*.” (CCC observation).

In this specific example, the issue was subsequently dropped and not included in the final CCC report, although the CCC member who brought up the issue was still encouraged to share his/her opinion with the trainee directly.

### Mechanisms for managing previously undocumented data

Recognizing the potential danger of previously undocumented data to exert undue or inappropriate influence on the group, there were several mechanisms that appeared to be invoked to ensure that such data were situated and managed. Perhaps the most obvious of these mechanisms was the structure of the discussion, which explicitly separated the formal presentation of the documentation from the relatively unstructured group discussion of the trainee. Because previously undocumented data were built into the framework of the discussion about each trainee, it was an expected and generally contained element of the meeting. However, there were other mechanisms that were observed (and explicitly mentioned in the interviews, suggesting that these mechanisms were invoked intentionally and reflectively).

#### Being mindful of who was providing previously undocumented data

During interviews, it was noted by committee members that if they had not worked with a trainee directly, they usually refrained from commenting beyond the formal data. This was corroborated by our observation, in which individuals who worked with a particular trainee less often tended to speak less about the trainee. Instead, these committee members offered a more outsider perspective, with suggestions of different ways to look at the situation (e.g., “could it just be his/her personality?”). Further, a committee member’s sources of information or reasons for offering previously undocumented data were often given as explicit qualifiers of authority, such as “I worked with him last month”, or “I met with her”. In this way, the group was able to self-monitor how much weight could and should be placed on a particular comment.

#### Being respectful of trainee confidentiality

Participants also noted in interviews that if personal circumstances might be relevant to a trainee’s current progress, they would ask the trainee prior to the CCC meetings how much the trainee was willing to have shared within the group. It was felt by participants that this would allow for a safe forum to discuss relevant contextual factors in interpreting a trainee’s progress.

#### Requiring a culture of direct feedback

The expectation of committee members giving direct feedback to trainees regarding any major issues prior to CCC meetings served as a mechanism to ensure that any previously undocumented data at the meeting would not be a surprise to the trainee. Participants perceived that learners would accept feedback derived from the CCC and delivered by the program director much better if the specific examples to develop and support the CCC-based feedback had already been discussed with the trainee by the faculty directly involved in the example. In the meeting itself, when an issue was raised for possible inclusion in the summary document, committee members were explicitly asked whether this was an issue they had discussed with the trainee. If not, they were encouraged to relay their individual assessments to the trainee directly, regardless of whether the issue was formally documented as part of the CCC.

#### By striving for consensus

The ultimate decision made about a trainee’s progress and what was documented from the meeting was summarized by either the chair or program director for collective agreement, with any previously undocumented data that were not widely experienced or agreed upon by the group being excluded from the final document. In this way, isolated incidents that did not resonate with the other members were relatively discounted, protecting the group from relying on one-off occurrences to make a high-stakes decision.

## Discussion

In our exploration of the use of previously undocumented data in the context of a CCC, we found that previously undocumented data were perceived by participants to be critical in providing a fair and holistic trainee assessment. Often, the formally documented information was seen as insufficient in detail and depth to allow a meaningful understanding of the central issues impacting a trainee’s progress. Such “problematic evidence” has also been reported in CCC meetings of other training programs, requiring more effortful interpretation by the group [23]. In the meeting itself, we observed that the utilization of discussions of previously undocumented data in a safe, regulated space allowed for synergistic group decision-making and problem-solving related to the diagnosis and management of a trainee’s trajectory. This combination of individual impressions to collectively build a group impression of a trainee was strongly suggestive of a co-constructivist approach to decision-making, in which the group strived for a shared understanding by integrating collective knowledge [24]. We did not see previously undocumented data functioning as an independent data source to be weighted, but rather as a nuancing process that contributed to the type of feedback that would be given to help the trainee improve. If the goal of trainee assessments is to be learner-centred and developmentally focused [4], our findings suggest that the responsible and appropriate application of previously undocumented data may well be essential to advancing this objective.

It might be argued that many of the examples of previously undocumented data raised in our results section could have been (or should have been) documented at that time of the observation. While increasing documentation should be encouraged, several factors raised by participants suggest that this might not be a reasonable expectation. Consistent with previous literature, participants expressed multiple rater barriers such as concerns about documenting what might be idiosyncratic experiences in permanent records [19] as well as limited time to provide meaningful documented assessments. Thus, our participants raised doubts about whether documented data could ever create a complete picture of the trainee. Less tangible issues, such as professionalism [25] and environmental/contextual considerations [26], have been found to affect overall resident assessment, but may be more challenging to document comprehensively. In short, documented assessments might indeed be further enhanced; however, a brief review of the literature supports our participants’ assertion that preceptors will continue to resist such requests for a variety of reasons. Therefore, the value of previously undocumented data in group discussions may well be irreplaceable. It is also worth noting that undocumented data can become documented within the CCC report. The best way to enact this is beyond the scope of our study but remains another important area of future focus.

While previously undocumented data were valuable in supporting the co-construction of a richer assessment profile, we recognize that this type of data can also potentially be misused or have unintended negative consequences. Potential cognitive biases during CCC deliberations have been described, such as: selection bias (relying on partial non-representative data), visceral bias (being overly influenced by emotions), availability bias (relying on more recent or memorable data) and groupthink (overreliance on group consensus) to name a few [9]. Our study, however, highlighted various mechanisms that have evolved in the longstanding CCC under study that allowed for the responsible treatment of previously undocumented data. Interestingly, even hearsay, which is most likely to be considered unacceptable in high-stakes decisions without further verification, was not observed to negatively distort the assessment of trainees in our study. In one instance, it was used to further enhance an impression and, in another instance, it was to address an aspect of training (i.e., teaching junior trainees) that could not have been fairly commented on by those present in the room. The latter instance highlights the importance of multi-source feedback and diverse CCC membership to be able to encompass multiple aspects of training, which may ultimately mitigate the use of hearsay wherever possible. Regardless, these protective mechanisms to optimize a fair and comprehensive trainee assessment would be important considerations for all other CCCs utilizing previously undocumented data.

The transferability of our study findings might be limited by several design factors. Although participants indicated during the interviews that the CCC meeting observed was fairly representative of how these meetings generally transpire, we cannot draw any conclusions regarding how this particular CCC may have utilized previously undocumented data differently in the deliberations of learners-in-difficulty. Being a longstanding group with minimal turnover means that the CCC members have had a substantial opportunity to develop and refine their group functioning. As such, this CCC may experience or be protected against different biases compared with newly created CCCs. Being a smaller training program means that almost all members of the CCC have worked with almost all trainees in the program. In larger programs, it may not be practically feasible for CCC membership to contain individuals who have all worked with every trainee directly. Thus, future research might well focus on CCCs of various types—small and large, old and new, procedural and non-procedural—to corroborate and extend the findings from our study. Future research directions may also explore how trainees perceive the use of previously undocumented data in their CCC assessments, as trainees are a major stakeholder of the decisions generated from this assessment approach. We also note that although we get the sense that the later interviews were reinforcing the responses of the earlier participants rather than offering substantial new insights, our limited sample size did not allow us to use saturation as a criterion for ceasing data collection.

Despite the potential limitations, this study suggests that, at least under some circumstances, previously undocumented data may play a vital and positive role in CCC conversations to create meaningful, developmental assessments that cannot be achieved by formally documented data alone. If so, the outright admonishment of previously undocumented data is a problematic construction and instead, mechanisms to utilize this type of data responsibly and appropriately should be advocated. With thoughtful incorporation of previously undocumented data as an essential part of the CCC assessment process, the group may more effectively work toward co-construction of a developmentally focused and learner-centred assessment.

## Caption Electronic Supplementary Material

Appendix 1
