# Supplementary material for: The role of previously undocumented data in the assessment of medical trainees in clinical competency committees
Source: Perspect Med Educ. 2020 Oct 6;9(5):286–93. doi: 10.1007/s40037-020-00624-x (PMC7550499; doi:10.1007/s40037-020-00624-x)
Supplement: Supplementary file 1 — Appendix 1 [file 40037_2020_624_MOESM1_ESM.docx]

**Appendix 1**

*Note: To match the language used by interview participants, the following synonyms were used:*

- *“P&P meetings” is used to mean the equivalent of “CCC meetings”*
- *“POWER evaluations” are used to mean the equivalent of “in-training evaluation reports (ITERs)”*

**Interview Guide**

**Introduction**:  Thank you for participating in our research. We are studying the Progress & Promotions (P&P) meetings that happen in the Division, which shares similarities to the Competence Committee meetings that will be implemented by all post-graduate medical training programs across Canada. We are trying to understand how these meetings help with the assessment of trainee performance. Please feel free to share stories and specific examples as much as possible. This interview will be audio-recorded and transcribed and completely de-identified prior to analysis. Please feel free to stop us if you feel uncomfortable at any time. Do you have any questions for us before we start?

1. WARM UP QUESTIONS: The P&P meetings have been happening in the division since 2008.
   1. Since when have you been attending these meetings? (i.e., what year?)
   2. Was the meeting that we observed on Jan 8, 2019 was similar to a typical P&P meeting? Why/why not?
   3. Describe any “rules” that you perceive associated with this meeting in general. What is the “tone” for discussing trainee progress that has been set up by the chair/group? How have you come to appreciate these rules (e.g., how did you know them? Does it facilitate discussion or not?)?
   4. Do you feel that these meetings, regardless of the outcome, is helpful to the trainee?
   5. Compared with POWER evaluations, how is the P&P meeting different? How is it similar?
2. INFORMATION UTILIZATION: We are interested in how the documented data presented for each fellow, such as POWER evaluations and exam scores, are used with the personal experiences with the fellow that individual faculty bring to the conversation during P&P discussions.
   1. What is your overall sense of how that works in the P&P meeting? Are there rules about when and what sorts of experiential information people are allowed to bring up in the discussion? Can you share your overall sense of how that works or when it goes wrong?
   2. How do you decide yourself what sort of information to share?
      1. For example, are there things you might share with another colleague informally about a resident in informal conversations that you might not share in the P&P? Do you have any specific examples? Why would you not share that in the P&P?
      2. What kinds of things would you share in the P&P?
   3. How do you think discussing a trainee’s personal circumstances (e.g. personal, stage of training, language/culture, personality) affects decisions about the trainee’s progress?
   4. How do you think discussing staff members’ personal experiences working with the trainee affects decisions about the trainee’s progress?
   5. How do you think discussing documented objective scores (e.g. POWER evaluation scores, exam scores) affect decisions about the trainee’s progress?
      1. Would using these alone be sufficient to gauge a trainee’s progress? Why/why not?
      2. E.g. Trainee F had no documented ITER scores – how did this impact how you thought about the information presented?
   6. Tell us about a time when you didn’t feel you had as much personal interaction with a trainee as others in the room - what information/tools did you use to form your opinion on the trainee? (e.g., someone else’s opinion? Scores?)
   7. What happens when two people disagree about how a trainee is performing during the meeting? Can you tell us about an experience when you disagreed about information presented (documented or undocumented) about a trainee’s progress? What happened?
   8. Can you think of a time when your opinion of a trainee changed because of something that you learned at the meeting, either about a trainee’s personal circumstances or a staff members’ personal experiences working with the trainee?
3. “WHY” PREVIOUSLY UNDOCUMENTED INFORMATION WAS BROUGHT UP:
   During the P&P meeting observed, you mentioned: [specific example(s)]
   1. What made you bring this up? (i.e., for what purpose? To support or to refute?)
4. INFORMAL DATA OUTSIDE THE P&P: Often, the progress of trainees is discussed informally with colleagues outside of P&P meetings. Can you think of an example when this happened?
   1. In what context?
   2. What did you discuss?
   3. Have these conversations influenced you in any way when making decisions about the trainee’s progress? Have these conversations influenced what topics/issues that you brought up in the P&P meeting (but was previously undocumented?)?
   4. Can you think of an example of something that you discussed with a colleague that you did not or would not bring up in the P&P meeting? Why/why not?
5. INFO UTILIZATION FOR TRAINEES IN DIFFICULTY: All the trainees discussed at the meeting observed appear to be progressing relatively well. Please think back to a P&P meeting when there was a trainee in difficulty.
   1. What information was used to reach the decision that the trainee was in difficulty (e.g. documented data vs previously undocumented data)?
   2. What factors played into decision (e.g. language barriers, international medical graduates, extenuating personal circumstances, etc.)?
   3. How well do you feel the P&P meeting identifies trainees in difficulty? Why?
6. WITHHELD INFORMATION: Sometimes there can be barriers (e.g. feeling intimidated, feeling like it is inappropriate) to sharing certain information with the rest of the group.
   1. Can you think of an example of when this happened to you during a P&P meeting?
   2. Was there any information that you didn’t bring up about the trainee’s progress at the observed P&P meeting? Why/why not?
   3. Do you have any thoughts/concerns about trainees who weren’t well represented in a P&P meeting? (or were described in a way that glossed over things/underrepresented the trainee?)
   4. Have you ever had a situation in which you felt that you had concerns or thoughts about a resident that were not well represented in the discussion? Why do you think that was [did you feel unable to share your concerns for any reason]?
7. UNINTENDED CONSEQUENCES: How do you feel about the ability to speak outside of documented objective information at the P&P meetings? (Are you comforted/reassured or troubled by this?)
   1. Was there a time that you felt like you needed to defend a trainee because of your role (i.e. mentor) with the trainee? (“saving face”)
   2. Was there anything that you heard about the trainee at a P&P meeting that changed how you subsequently interacted with or viewed the trainee in the future?
   3. Have you ever felt that some of the things that were brought up about a resident were inappropriate or might have done damage to the overall opinion of the resident? How was that handled?
